# Treatment of Rectovaginal Fistula Using Sphincteroplasty and Fistulectomy

**DOI:** 10.1155/2018/5298214

**Published:** 2018-05-06

**Authors:** Kemal Beksac, Atakan Tanacan, Nejat Ozgul, Mehmet Sinan Beksac

**Affiliations:** ^1^Department of General Surgery, Ankara Oncology Hospital, Ankara, Turkey; ^2^Department of Obstetrics and Gynecology, Hacettepe University, Ankara, Turkey

## Abstract

**Aim:**

To assess the results of the treatment of rectovaginal fistulas with incontinence and impaired anal tonus.

**Materials and Methods:**

This study comprised three rectovaginal fistula groups that were treated using sphincteroplasty and fistulectomy: group 1: eight women with simple rectovaginal fistula due to birth trauma; group 2: six rectovaginal fistula cases that were associated with chronic inflammatory diseases; and group 3: five cases with at least one failed repair attempt. In the second step, operations that took place before the year 2000 were compared to the operations that took place after the year 2000 in terms of demographic and clinical characteristics.

**Results:**

All of the simple rectovaginal fistula cases healed after the operation. Five of the group 2 patients healed after the operation. However, 1 patient with Crohn's disease needed to undergo reoperation, but successfully healed after 6 months. On the contrary, 3 patients in group 3 healed (60%) whereas 2 of them failed to heal. Clinical characteristics of the patients were different between the groups (before and after the year 2000).

**Conclusion:**

The choice of operation must be done according to the patient's underlying pathology. Proper management of associated inflammatory diseases and systemic disorders is recommended for necessary complex cases.

## 1. Introduction

Successful treatment of the rectovaginal fistula is dependent on the etiology, size of the fistula, location, status of the sphincter, and associated systemic disorders, such as diabetes mellitus and autoimmune disorders [1–5]. The experience of the surgeon and the previous attempts at repair are also important factors of the success rate of the operations [6, 7].

Simple rectovaginal fistulas are defined as fistulas located in the low-to-mid portion of the vaginal septum, less than 2–3 cm in diameter, and are secondary to birth trauma [8]. The success rate in simple cases is high, while outcomes of other complicated cases are comparatively poorer depending on the etiology and the presence of associated systemic diseases [1, 2]. A wide spectrum of etiological risk factors were described for the occurrence of rectovaginal fistulas [7, 9]. Previous failed surgical attempts at rectovaginal fistula repair make future operations more complex [7, 10].

There are various operative procedures, such as the advancement flap, sphincteroplasty and fistulectomy, coloanal anastomosis, and gracilis muscle repair to manage rectovaginal fistulas [11, 12]. The choice of the operative technique greatly depends on the status of the fistula and the etiological rationale behind this medical complication [1, 11].

In this study, we have compared the outcomes of the sphincteroplasty and fistulectomy procedures in three different rectovaginal fistula groups, such as simple rectovaginal fistula, rectovaginal fistulas associated with chronic inflammatory and autoimmune disease, and cases with at least one failed repair attempt together with inflammatory diseases. All patients were incontinent and felt to have impaired anal tonus.

## 2. Materials and Methods

This retrospective study comprised three rectovaginal fistula groups that were treated by sphincteroplasty and fistulectomy: group 1: 8 female patients with simple rectovaginal fistula due to birth trauma; group 2: 6 rectovaginal fistula cases that were associated with chronic inflammatory diseases (4 Crohn's disease, 1 ulcerative colitis, and 1 Behçet's disease); and group 3: 5 cases with at least one failed repair attempt (4 Crohn's disease and 1 systemic lupus erythematosus).  Table 1 shows the clinical features and the risk factors of the study groups.

Patients were evaluated in terms of bowel habit, dietary intake, medications, associated medical problems (especially chronic inflammatory diseases, diabetes mellitus, and autoimmune disorders), and family histories. Vaginal and rectal examinations were performed in order to evaluate vulva, vagina, and anorectal region together with the characteristics of the fistula. Resting pressure and voluntary contractions of the anal sphincter complex and puborectalis muscle were assessed.

All patients included to this study were incontinent of either flatus or stool and were felt to have relaxed anal tonus, and all of them had fecal incontinence at a level that would impair their quality of life. We could not use Cleveland Clinic Incontinence Score (CCIS) or Fecal Incontinence Severity Index (FISI) for further evaluation of the fecal incontinence in all cases due to the retrospective design of the study [13]. Preoperative bowel preparation was done by prescribing the patients a clear liquid diet three days before the surgery. Enema was applied at the night before the surgery.

**Figure 1:**
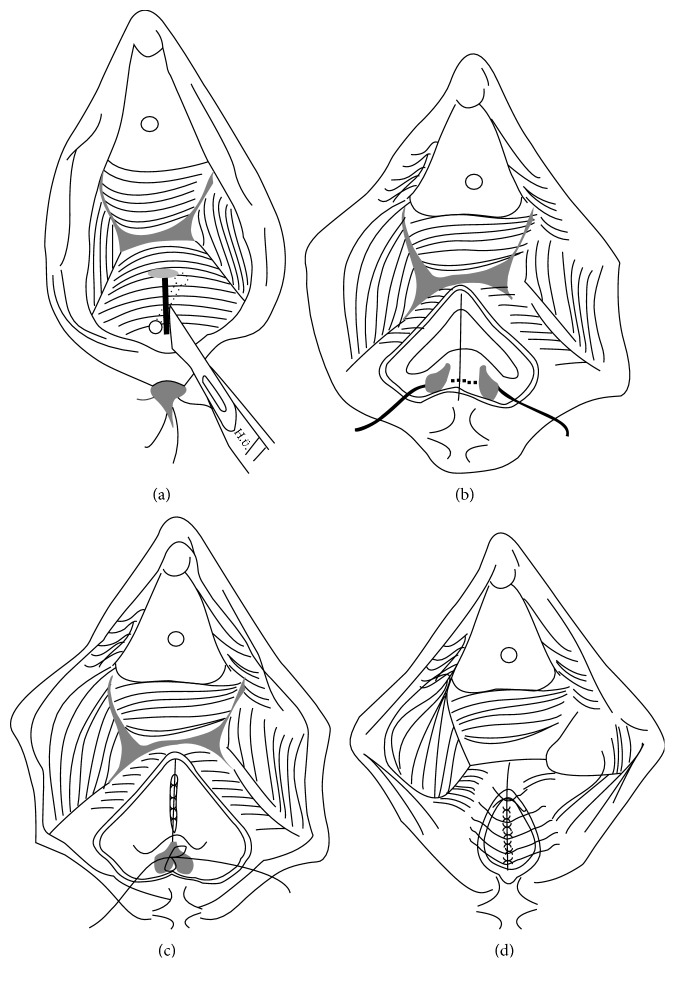
(a) A curvilinear incision was made anterior to the rectum and a plane between the rectum and vagina was created. Then, identification and excision of the fistula tract was performed. (b) Next step is the preparation of the anal sphincter complex. (c) Visualized sphincter muscles were fixed anteriorly after the repairment of rectoanal mucosa. (d) Then, operation was completed after the restoration of the vaginal mucosa in a similar manner as episiotomy.

Sphincteroplasty and fistulectomy were used in all cases [14]. A curvilinear incision was made anterior to the rectum and a plane between the rectum and vagina was created. Then, identification and excision of the fistula tract were performed together with the preparation of the anal sphincter complex. Visualized sphincter muscles were fixed anteriorly after the repairment of rectoanal mucosa. Then, operation was completed after the restoration of the vaginal mucosa in a similar manner as episiotomy (Figure 1). 4-0, 1-0, and 3-0 absorbable sutures (polyglactin 910) were used for rectoanal mucosa repairment, anal sphincter fixation, and vaginal mucosa restoration, respectively. We did not use drain in our cases but our department protocol considers drains in necessary cases.

Soft, formed, deformable stool was provided in the postoperative period at least for two weeks with the help of clear liquid diet, copious fluid intake, and the use of stool softeners. Oral broad-spectrum antibiotic therapy was given for 3–5 days postoperatively. Sexual activity or any physical activities more strenuous than a slow walk were avoided by the patients for three weeks after the surgery. Cases that required other operations were excluded from the study. Subsequently, operations that took place before the year 2000 were compared with operations which took place after the year 2000 in terms of demographic and clinical characteristics.

Statistical analyses were performed using the SPSS software version 22. The variables were investigated using visual (histograms and probability plots) and analytical methods (Kolmogorov–Smirnov/Shapiro–Wilk's test) to determine whether they were normally distributed. Descriptive analyses were presented using medians, minimum-maximum values for the non-normally distributed data, and ordinal variables. Since some variables were not normally distributed, nonparametric tests were conducted to compare these parameters, as well as to compare the ordinal variables. The Mann–Whitney *U* test was used to evaluate the medians between the groups. A proportion of the patients were presented using cross tabulations. The chi-square test or Fisher exact test (when chi-square test assumptions do not hold due to low expected cell counts), where appropriate, was used to compare these proportions in different groups. A *p* value of less than 0.05 was considered to show a statistically significant result.

This study was approved by the Hacettepe University Ethics Committee (no = GO 16/101-30). Informed consent forms were obtained from the patients before surgical interventions.

## 3. Results

All of the simple rectovaginal fistula cases (group 1) healed after the operation. Five of the group 2 patients healed after the operation. However, 1 patient with Crohn's disease underwent another operation, but recovered successfully after 6 months. On the contrary, 3 patients in group 3 healed (60%) and 2 of them failed to heal.

Diabetes mellitus was defined in 2 and 3 cases in group 1 and group 3, respectively. In all cases, patients underwent operation after careful management and control of the associated systemic disorders (metabolic and immunological).  Table 2 shows the clinical features of the patients who underwent operation before and after the year 2000. It has been demonstrated that the clinical characteristics of the patients were different among groups in terms of age, presence of maternal immune disorders (especially chronic inflammatory disease such as Crohn's disease), presence of birth trauma, and previous failed fistula operations (*p*=0.016, <0.001, <0.001, and  0.026, resp.). The “after 2000 patients” were older and almost all of them had inflammatory bowel diseases or other autoimmune disorders, while rectovaginal fistulas of the “before 2000 patients” were mainly due to birth trauma. We have also shown that 45.5% (5/11) of the “after 2000 patients” had at least one previous failed rectovaginal fistula operation.

## 4. Discussion

Rectovaginal fistula is one of the most complicated subjects in obstetrics and gynecology practice. It has a devastating effect on patients' quality of life and appropriate management of this complication is challenging for the physicians [15]. Obstetrical traumas, inflammatory bowel diseases, carcinomas, radiotherapy sequels, infectious processes, and postsurgical complications were the main etiological factors of rectovaginal fistula [15]. However, there is still no consensus on the most effective treatment option for this condition as recurrence rate is significantly high in all procedures [16]. Surgery is necessitated in most of the cases, although, conservative management may be applied in some patients with mild symptoms [10, 15].

Etiology of the rectovaginal fistula, its location and size, the quality of the surrounding tissue, associated systemic disorders, previous attempts at repair, and the experience of the surgeon determine the surgical approach [17]. Mucosal advancement flap repair, transanal sleeve advancement flap, perineoproctectomy with layered closure, sphincteroplasty and fistulectomy, coloanal anastomosis, and gracilis muscle repair are the main surgical protocols used for the treatment of rectovaginal fistulas [15]. Biological agents such as fibrin glue and surgisis plug may also be applied in some suitable cases [15]. Diverting colostomy may be necessitated in the most complicated cases like radiation-induced fistulas and fistulas secondary to inflammatory bowel diseases [18]. Stem cell therapy for the repair of rectovaginal fistulas has been investigated in various studies and promising results were reported [19–22].

Results of the endorectal advancement flap when used as a primary procedure in the treatment of simple rectovaginal fistula was reported to be acceptable [23, 24]. However, complicated cases required a more complicated approach [19, 25]. In this study, patients with incontinence and impaired anal tonus were operated by sphincteroplasty and fistulectomy. Our success rate was ideal for simple rectovaginal fistula cases (all of them due to birth trauma). However, cases with associated chronic inflammatory diseases and cases with previous failed fistula repair attempts needed further medical attention. Three failed operations occurred in groups 2 and 3. We believe that it is not only the technique itself and the experience of the surgeon to enable a smoother procedure but also the control of immunologic/metabolic inflammation. Especially, persistent cases require complicated techniques and approaches to achieve better results [10, 26]. Stem cell therapy and conservative approaches might also be the choice of management in such cases [19].

Nowadays, “birth-trauma-related rectovaginal fistula” is less frequent most likely because of the changes in obstetrics practice [27, 28]. Increased cesarean section rates and decreased operative vaginal deliveries might be the reason for this change [29, 30]. In this study, it is shown that today's patient profile is different compared to previous decades in terms of the etiology. Almost all of our recent rectovaginal fistula cases were complications of inflammatory bowel diseases, but not birth trauma. A limitation of this study is the small number of cases and the patient profile of our institution.

In conclusion, the choice of operation must be determined according to the patient's underlying pathology. In view of the poor rates of healing, proper management of associated inflammatory diseases and systemic disorders is recommended for complex cases and for those who have previously undergone an operation. The forthcoming problem seems to be the presence of less experienced surgeons and more complicated cases.

## Figures and Tables

**Table 1:** Clinical features and risk factors of birth-related trauma, inflammatory bowel disease, and previous failed operations of the rectovaginal fistula groups (means, standard deviations, and percentages).

Rectovaginal fistula group	Birth trauma (*n*=8)	Inflammatory bowel disease (*n*=6)	Previous failed operation (*n*=5)
Age (years)	27.63 ± 7.17	36.33 ± 7.47	42 ± 9.08
Gravida	2.75 ± 1.90	1.67 ± 0.81	2.00 ± 1.00
Parity	1.63 ± 1.18	0.83 ± 0.40	1.20 ± 0.44
Maternal disease	Asthma (*n*=1), Hashimoto thyroiditis (*n*=1), diabetes mellitus (*n*=2)	Crohn's disease (*n*=4), ulcerative colitis (*n*=1), Behçet's disease (*n*=1)	Crohn's disease (*n*=4), SLE/Sjogren's syndrome (*n*=1), diabetes mellitus (*n*=3)
Operation success	8/8 (100%)	5/6 (83.3%)	3/5 (60%)

**Table 2:** Clinical features of the patients who underwent operation before and after the year 2000 (medians, minimum-maximum values, and percentages together with the *p* values).

Time period	Before 2000 (*n*=8)	2000 and later (*n*=11)	*p* value
Age (years)	26 (18 to 38)	36 (27 to 50)	0.016
Gravida	2 (1 to 6)	2 (1 to 3)	0.338
Parity	1 (1 to 4)	1 (0 to 2)	0.203
Previous RVF operation (%)	0/8 (0%)	5/11 (45.5%)	0.026
Maternal autoimmune disease (%)	2/8 (25%)	11/11 (100%)	<0.001
Birth trauma (%)	8/8 (100%)	2/11 (18.2%)	<0.001
Operation success (%)	8/8 (100%)	8/11 (72.7%)	0.107
Diabetes (%)	2/8 (25%)	3/11 (27.2%)	0.719

RVF: Rectovaginal fistula.

## References

[1] Pinto R. A., Peterson T. V., Shawki S., Davila G. W., Wexner S. D. (2010). Are there predictors of outcome following rectovaginal fistula repair?.

[2] Penninckx F., Moneghini D., D’Hoore A., Wyndaele J., Coremans G., Rutgeerts P. (2001). Success and failure after repair of rectovaginal fistula in Crohn’s disease: analysis of prognostic factors.

[3] Arrowsmith S. D., Barone M. A., Ruminjo J. (2013). Outcomes in obstetric fistula care: a literature review.

[4] Brown H. W., Wang L., Bunker C. H., Lowder J. L. (2012). Lower reproductive tract fistula repairs in inpatient US women, 1979–2006.

[5] Beksac K., Turgal M., Basaran D., Aran O., Beksac M. S. (2014). Vaginoperineal fistula as a complication of perianal surgery in a patient with Sjögren’s syndrome: a case report.

[6] Rahman M., Al-Suleiman S., El-Yahia A., Rahman J. (2003). Surgical treatment of rectovaginal fistula of obstetric origin: a review of 15 years’ experience in a teaching hospital.

[7] Halverson A. L., Hull T. L., Fazio V. W., Church J., Hammel J., Floruta C. (2001). Repair of recurrent rectovaginal fistulas.

[8] Baig M. K., Zhao R. H., Yuen C. (2000). Simple rectovaginal fistulas.

[9] Jordán J., Roig J., García-Armengol J., García-Granero E., Solana A., Lledó S. (2010). Risk factors for recurrence and incontinence after anal fistula surgery.

[10] Lawson E. H., Roberts P. L. (2017). Benign anal disease: management of the recurrent anovaginal/rectovaginal fistula.

[11] Abu Gazala M., Wexner S. D. (2017). Management of rectovaginal fistulas and patient outcome.

[12] Marecik S., Abcarian A. M., Prasad L. M. (2017). Complications of rectovaginal fistula repair.

[13] Hayden D. M., Weiss E. G. (2011). Fecal incontinence: etiology, evaluation, and treatment.

[14] MacRae H. M., McLeod R. S., Cohen Z., Stern H., Reznick R. (1995). Treatment of rectovaginal fistulas that has failed previous repair attempts.

[15] Rivadeneira D. E., Ruffo B., Amrani S., Salinas C. (2007). Rectovaginal fistulas: current surgical management.

[16] Ratto C., Litta F., Donisi L., Parello A. (2015). Fistulotomy or fistulectomy and primary sphincteroplasty for anal fistula (FIPS): a systematic review.

[17] Göttgens K. W., Smeets R. R., Stassen L. P., Beets G., Breukink S. O. (2014). The disappointing quality of published studies on operative techniques for rectovaginal fistulas: a blueprint for a prospective multi-institutional study.

[18] Sher M. E., Bauer J. J., Gelernt I. (1991). Surgical repair of rectovaginal fistulas in patients with Crohn’s disease: transvaginal approach.

[19] Garcia-Olmo D., Herreros D., Pascual I. (2009). Expanded adipose-derived stem cells for the treatment of complex perianal fistula: a phase II clinical trial.

[20] Guadalajara H., Herreros D., De-La-Quintana P., Trebol J., Garcia-Arranz M., Garcia-Olmo D. (2012). Long-term follow-up of patients undergoing adipose-derived adult stem cell administration to treat complex perianal fistulas.

[21] Garcia-Olmo D., Guadalajara H., Rubio-Perez I., Herreros M. D., de-la-Quintana P., Garcia-Arranz M. (2015). Recurrent anal fistulae: limited surgery supported by stem cells.

[22] Ibraheim H., Giacomini C., Kassam Z., Dazzi F., Powell N. (2018). Advances in mesenchymal stromal cell therapy in the management of Crohn’s disease.

[23] Sonoda T., Hull T., Piedmonte M. R., Fazio V. W. (2002). Outcomes of primary repair of anorectal and rectovaginal fistulas using the endorectal advancement flap.

[24] Mizrahi N., Wexner S. D., Zmora O. (2002). Endorectal advancement flap.

[25] Jarrar A., Church J. (2011). Advancement flap repair: a good option for complex anorectal fistulas.

[26] Wang D., Chen J., Zhu L., Sang M., Yu F., Zhou Q. (2017). Surgical repair of rectovaginal fistula using the modified martius procedure: a step by step guide.

[27] Wall L. L. (2006). Obstetric vesicovaginal fistula as an international public-health problem.

[28] Betran A., Torloni M., Zhang J., Gülmezoglu A. (2016). WHO Statement on caesarean section rates.

[29] Miller L. A., Mills E., Baja A., Clarke B., Zarlengo G. (2016). An analysis of operative delivery practices over 30 years [11D].

[30] Balcı O., Özdemir S., Mahmoud A., Acar A. (2010). Birth rates and cesarean indications at Selçuk University during five-year period.

